# Evaluation of airway obstruction by nasopharyngoscopy: comparison of the Müller maneuver versus induced sleep

**DOI:** 10.1016/S1808-8694(15)30121-X

**Published:** 2015-10-19

**Authors:** Marcelo Gervilla Gregório, Márcia Jacomelli, Adelaide C. Figueiredo, Michel Burihan Cahali, Wilson Leite Pedreira Junior, Geraldo Lorenzi Filho

**Affiliations:** a PhD, Assistant physician at the Respiratory Endoscopy Service of the University of São Paulo Medical School; b PhD, Assistant physician at the Respiratory Endoscopy Service of the University of São Paulo Medical School; c PhD Student, Pneumology - University of São Paulo Medical School - Polysomnography technician - Instituto do Coração, INCOR; d PhD. Assistant at the Department of otorhinolaryngology - University of São Paulo Medical School; e PhD. Head of the Department of Pneumology of the Fleury Laboratory; f Associate Professor, Head of the Sleep Laboratory - Incor, Discipline of Pneumology - Department of Cardio-Pneumology. University of São Paulo Medical School - Hospital

**Keywords:** apnea, laryngoscopy, midazolam, sleep

## Abstract

The use of nasopharyngoscopy during the application of intrathoracic pressure (Müller maneuver) is frequently employed to establish the site of upper airway obstruction. The Müller maneuver, however, is used when the patient is awake and therefore may not correlate with obstruction occurring during sleep. **Aim:** to compare the degree of pharyngeal obstruction in the retropalatal and retroglossal regions during the Müller maneuver versus induced sleep using nasopharyngoscopy. **Study design:** A prospective, case series study. **Material and methods:** Eight patients (three males, five females), with a mean age of 48.6 +/- 9,2 year, underwent nasopharyngoscopy to assess airway anatomy and funciton during the Müller maneuver while awake and during sleep induced by drip infusion of Midazolam. **Results:** Retropalatal obstruction was similar during the Müller maneuver and sleep (mean + standard deviation = 3.13 +/- 0.99 and 2.75 +/- 0.46, p= 0.234). Retroglossal obstruction was significantly lower during Müller maneuver compared to sleep (mean + standard deviation 0.63 +/- 1.06 and 2.63 +/- 1.30, respectively, p= 0.005). **Conclusion:** The sleep inducing method was safe under the monitored conditions of this study, and detected more retroglossal obstruction than the Müller maneuver.

## INTRODUCTION

Snoring and obstructive sleep apnea are very common in the general population and usually happen because of a partial or total collapse of the upper airways during sleep1. Upper airway segments responsible for airflow resistance during sleep involve the nasal cavities (such as nasal conchae hypertrophy, septum deviation and polyps), the rhinopharynx (adenoid hypertrophy and flaccid palate), the oropharynx (lateral pharynx wall hypertrophy and large uvula, palatine tonsils hypertrophy or lingual tonsil hypertrophy); or pharyngeal-Larynx (tumors and malacia), representing different places of obstruction. Assessment by indirect nasal-pharyngoscopy is carried out routinely, because these anatomical alterations are responsible for an increase in airflow resistance and are potentially correctable by surgery.

The obstruction functional or dynamic components are represented by collapse and pharyngeal walls narrowing or vibration during sleep, and may be more evident in isolate or multiple segments (retropalatal and/or retrolingual regions). Muscle tone, airway inner diameter, fat deposits and cranial-facial bone shape, all influence the degree of obstruction in these segments of the soft pharynx, which do not have bone or cartilage support^2^, ^3^, ^4^. In these matters, the nasal-pharyngoscopy with Müller’s maneuver (which assesses the dynamic behavior and the degree of retropalatal and retrolingual collapse during maximum inspiration with both the mouth and he nose occluded) is the assessment method currently being used5. Nonetheless, its usefulness in surgery planning with the goal of correlating its findings with the success expectations of surgical treatment (especially, uvulopalatopharyngoplasty - UPPP) is extremely controversial^6^, ^7^, ^8^. There are some papers, which correlate retropalatal collapse alone with the therapeutic success of UPPP for the treatment of OSAS, and others did not find this correlation^9^, ^10^.

There are numerous potential limitations associated with Müller’s maneuver: it assesses the upper airways during the day, while obstruction happens during sleep. Another limitation is that most of the studies do not quantify the negative pressure generated by the patient during Müller’s maneuver. In order to reduce these biases, some studies with videonasal-laryngoscopy under sleep induction have been carried out since 1990^11^, ^12^. These studies show differences between obstruction sites (retropalatal and retrolingual) observed during Müller’s maneuver^13^, ^14^ and during induced sleep, suggesting that this last one would be the method closer to what really happens during sleep.

The goal of the present investigation was to describe an anatomical and functional method (comparing the upper airways gauge variation in the retropalatal and retrolingual areas) through induced sleep and compare it to Müller’s maneuver carried out with the patient awake.

## MATERIALS AND METHODS

This research protocol (# 370/03) was approved by the Ethics Committee of our institution.

We studied 8 patients (3 men and 5 women), at the ages of (mean ± standard deviation) 48.6 ± 9.2 years and body mass index (BMI) of 26.6± 5.7 kg/m2 with clinical history of loud snoring. All the patients went through videonasal-pharyngoscopy by means of an OlympusR video bronchoscope of 4.9mm of external diameter. No topical anesthesia on the oropharynx or larynx was used. We used 2% lidocaine gel for nasal lubrication, facilitating the scope’s passage.

**Figure 1:**
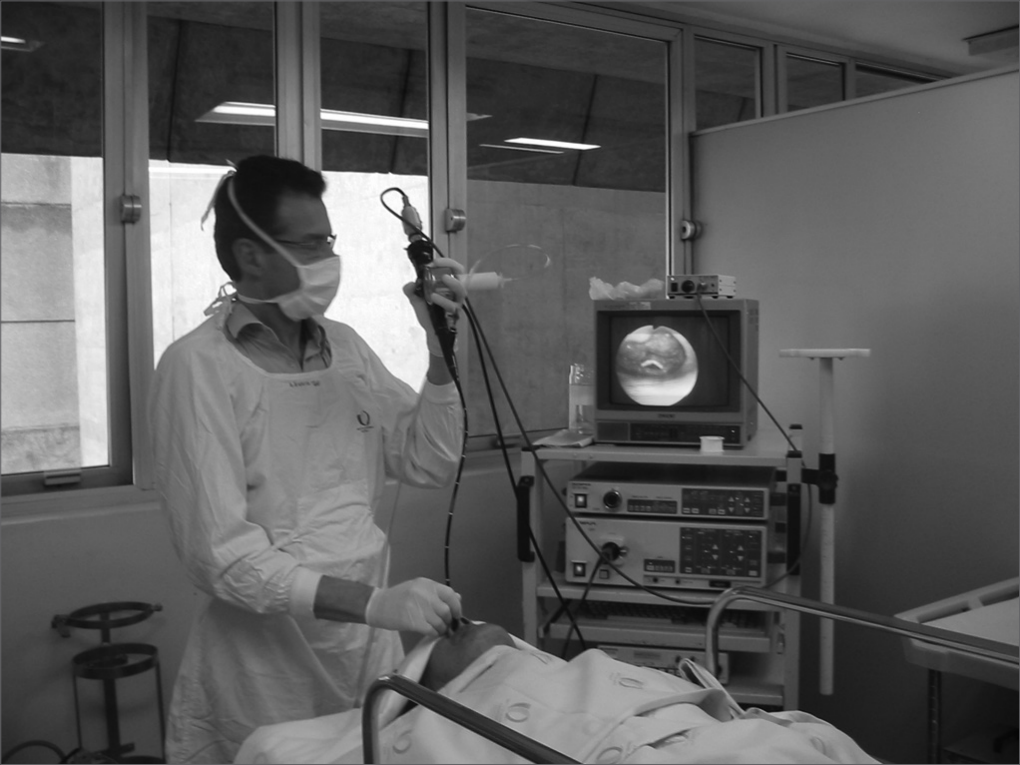
Müller’s maneuver through intermittent nasal occlusion and maximum inspiration. We used a manovacuometer for pressure recording.
c) induced sleep. Müller’s maneuver was based on creating negative pressure through vigorous inspiration having both the nose and the mouth occluded. We considered the maneuver to be effective only when the patient reached the negative pressure of 40 cm/H_2_O^14^, ^15^. For this maneuver we positioned the video bronchoscope in two different levels: at the rhinopharynx and at the oro-pharyngeal transition site, allowing us to quantify the collapse in these two levels, according to the description below. After Müller’s maneuver, we induced the patient’s sleep by dripping 10mg of midazolam diluted in 100mL of saline solution in the burette. We interrupted the endovenous infusion as soon as the patient slept - easily noticed after he/she started snoring. During induced sleep, we positioned the video bronchoscope in the same regions where we monitored the Müller’s maneuver (retropalatal and retrolingual). We monitored the patients by continuously recording their breathing by means of a nasal flow sensor (pressure canula), respiratory effort (thoracic-abdominal band) and pulse oxymeter (Stardust, RespironicsR device) during videonasal-pharyngoscopy, and for 15 minutes after removal of the fiberscope. These exams were carried out in the bronchoscopy room, equipped with material for intubation, mechanical ventilation and emergency care. Data obtained by polysomnography during videonasal-pharyngoscopy were later compared with those from the conventional polysomnography, and this comparison will be published later.

**Chart 1:**
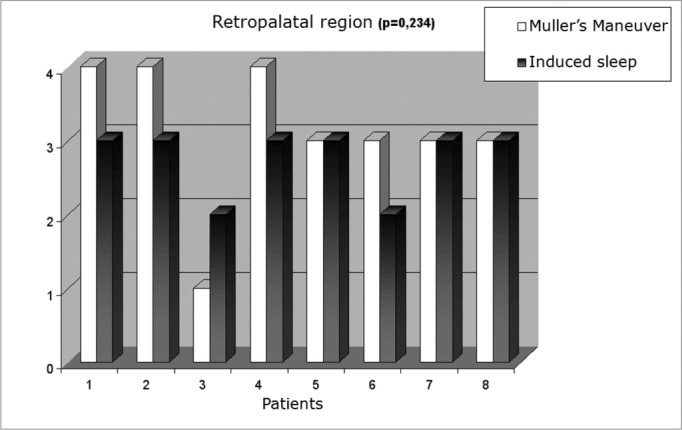
Obstruction levels seen during Müller’s maneuver and during induced sleep in the retropalatal and retrolingual areas

Videonasal-pharyngoscopy was carried out with the patients laying down on dorsal decubitus, with peripheral venous punction and monitoring. We did photographic documentation in three consecutive stages:

a) anatomical evaluation of the nose, pharynx and larynx;
b) Müller’s maneuver with the patient awake, in dorsal decubitus, measuring inspiratory pressure by means of a manovacuometer coupled to the bronchoscope’s working port (Figure 1);

The obstruction of the regions studied, both during Müller’s maneuver, as well as during induced sleep, was measured in a semi-quantitative fashion in: no obstruction (0) or up to 25% obstruction (1), 50% (2), 75% (3) and 100% (4) of obstruction. (Chart 1)

We used the Mann-Whitney Rank Sum Test (SigmastatÒ 3.1) in order to compare retropalatal and retrolingual obstruction seen during Müller’s maneuver and during induced sleep.

## RESULTS

Endoscopic anatomical evaluation showed nasal conchae hypertrophy in six of the eight patients (75%) and nasal septum deviation in four of the eight (50%). We did not see other anatomical alterations, including adenoid or tonsil hypertrophy, tumors or malformations. Midazolam dose for sleep induction varied from 4 to 10mg (mean value of 6.1 + 2.1). All patients awoke spontaneously after the study - it was not necessary to use drugs for pharmacological reversion.

**Figure 2:**
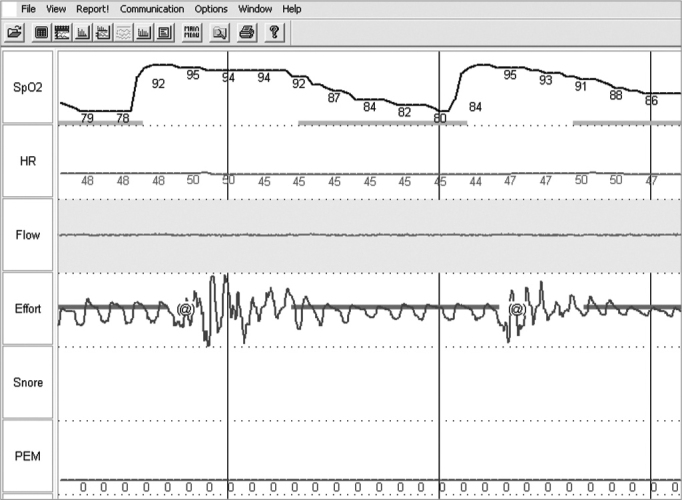
Polysomnography during nasal endoscopy under induced sleep.

Obstruction levels we saw during Müller’s maneuver and induced sleep were of mean + standard deviation 3.13 + 0.99 and 2.75 + 0.46, respectively, p= 0.234 (Graph I). In contrast, retrolingual obstruction was significantly less during Müller’s maneuver (mean + standard deviation 0.63 + 1.06 and 2.63 + 1.30, respectively, p= 0.005) (Graph II). Figure 2 shows respiratory monitoring during induced sleep of a patient who presented with severe obstructive sleep apnea. Monitoring showed that the respiratory events during induced sleep were obstruction-related (that is: there was inspiratory effort detected by the chest-band during breathing). The lower saturation observed was of 85%, present during obstructive events. No patient required oxygen complementation or sleep interruption because of prolonged apnea.

**Chart 2:**
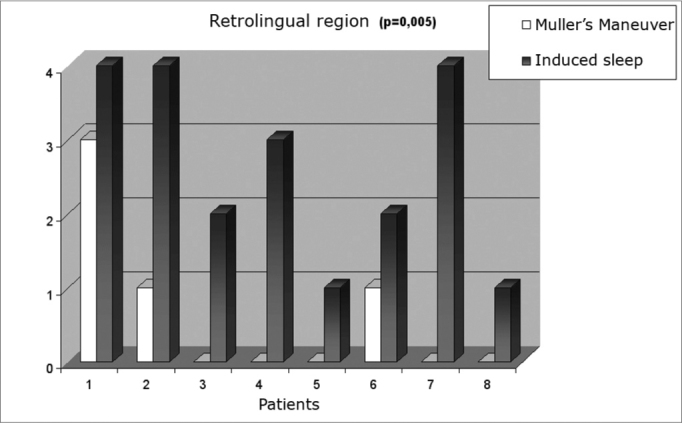
Retrolingual region collapse estimated by Müller’s maneuver and induced sleep.

## DISCUSSION

Our investigation described the videonasal-pharyngoscopic evaluation of the upper airway, in a sequential manner, allowing for diagnoses of structural (anatomical alterations) and functional disorders during the patients waking hours, by means of the Müller’s maneuver and during sleep induced by midazolam. While retropalatal obstruction was common with the two maneuvers, during induced sleep we noticed more retrolingual obstruction than Müller’s maneuver with the patient awake. Thus, these data suggest that Müller’s maneuver can underestimate retrolingual collapse. Midazolam-induced sleep may become an important tool in assessing the upper airway obstruction site, and in the future it may help in the therapeutic choice for these patients with snoring, in whom we suspect of sleep obstructive apnea syndrome.

On the way between the choanas and the hypopharynx, the retropalatal and retrolingual regions are the ones most prone to collapse16, since they are narrow and do not have a bony or cartilaginous support framework. Therefore, these regions seem to be the primary site responsible for partial collapse, causing snoring. Total collapse causes obstructive sleep apnea. Intra-thoracic negative pressure und resistance (Müller’s maneuver), associated with nasallaryngoscopy, allows for a dynamic evaluation of upper airway collapse in the entire pharynx.^17^ Some studies consider Müller’s maneuver a key point in the functional pre-operative assessment for uvulopalatopharyngoplasty in patients with snoring and/or obstructive sleep apnea syndrome. Müller’s maneuver has been used in the clinical practice not only to locate and classify retropalatal and retrolingual obstruction, but also to aid in surgical decision making. Obstruction alone has been considered a success factor for uvulopalatopharyngoplasty. Nonetheless, this concept has been broadly challenged, as well as the usefulness of traditional approaches^18^. Assessing the laxity of the entire larynx by means of the Müller’s maneuver has contributed to the planning of different palatal pharyngoplasties; however its role in locating the site of obstruction must be revised, since its quantification is not correlated with OSAS, nor with the results of different surgical treatments.^19^, ^20^ In greater or lesser degrees, OSAS must be considered a disease that involves the entire pharynx. In this context, we believe that the induced sleep method proposed in the present investigation may become an important tool to aid in this surgical approach.

In our small sample of patients, we found nasal alterations in a great number of patients. Nasal disease must be better described during the initial nasal endoscopy exam, in order to provide data regarding the degree of airflow obstruction. Nasal disease is common in the general population and it may play a role in pharyngeal obstruction in patients with sleep obstructive apnea syndrome^21^, ^22^, ^23^. Some authors suggest that nasal obstruction usually plays a role in the so called “oral breather”, in whom there is a posterior shift of the tongue and mandible, reducing the airway gauge and altering the efficacy of pharyngeal dilating muscles (predisposing the patient to collapse during sleep). These factors, associated with obesity, soft tissue hypertrophy (pharyngeal lateral walls) and cranial-facial structural alterations play a role in the obstruction of collapse-susceptible regions and increase in respiratory effort.

Our study included the measurement of the negative pressure generated by the patient during Müller’s maneuver, by means of a manovacuometer coupled to the working port of the videobronchoscope (Figure 1). Thus, we selected only the maneuvers that created significant effort (Pressure >40 cm H2O). This is a low cost methodology that is not routinely used and could be easily added to the method. In fact, we observed that many patients had to have the maneuver repeated many times before we could reach an acceptable minimal pressure. Most of the papers published so far did not use any type of objective measurement of the negative pressure created by the patient during Müller’s maneuver. This limitation to the method may contribute to explain the disagreeing results among studies.

Because of the limitations and controversies related to the real usefulness of laryngoscopy in waking patients, many authors advocate the idea that induced sleep could be a more accurate method to quantify and locate the point of collapse in the upper airway (UAW).^24^, ^25^, ^26^ For example, a prospective and controlled study14 which induced sleep with titrated doses of propafenone (propofolR) and evaluated UAW behavior in a group of patients with apnea showed that this method was safe, specific and sensitive. In our study we used a methodology similar to the one described by Sadaoka et al.^27^ and we induced sleep with low doses and slow dripping of midazolam, acting only as a sleep inducer and not as a sedative agent. We showed that the method is safe, and during the procedure the patients could wake up at any time. All patients woke up spontaneously after the test, without the need to use antagonist agents. Despite all of this, we highlight the importance of employing this method in a place that has the means for oro-tracheal intubation, mechanical ventilation and post-procedure observation should it be necessary. Midazolam has the advantage of being inexpensive and more readily available than propofol. In our country, to use propofol one needs to have an anesthesiologist to help, and this increases costs. In our study, nasal endoscopy with induced sleep was followed by respiratory monitoring in real time and allowed us to document the fact that the apneas seen were indeed obstructive (Figure 2).

## CONCLUSION

This study opens an important perspective in the preoperative endoscopic assessment of patients with snoring and obstructive sleep apnea, and we concluded that this estimate of the obstruction level by induced sleep is better than Müller’s maneuver.
